# Novel parvovirus in an outbreak of fatal enteritis in European hedgehogs (*Erinaceus europaeus*), Italy, 2022

**DOI:** 10.1128/spectrum.02494-23

**Published:** 2023-09-20

**Authors:** Gianvito Lanave, Georgia Diakoudi, Francesco Pellegrini, Roberto Lombardi, Michela Prioletti, Elena Circella, Antonio Camarda, Barbara Di Martino, Michele Camero, Nicola Decaro, Krisztián Bányai, Antonio Lavazza, Canio Buonavoglia, Vito Martella

**Affiliations:** 1 Department of Veterinary Medicine, University of Bari Aldo Moro, Bari, Italy; 2 Department of Veterinary Medicine, University of Teramo, Teramo, Italy; 3 National Laboratory of Infectious Animal Diseases, Antimicrobial Resistance, Veterinary Public Health and Food Chain Safety, Veterinary Medical Research Institute, Budapest, Hungary; 4 Department of Pharmacology and Toxicology, University of Veterinary Medicine, Budapest, Hungary; 5 Istituto Zooprofilattico Sperimentale della Lombardia e dell'Emilia Romagna, Brescia, Italy; University of Manitoba, Winnipeg, Manitoba, Canada

**Keywords:** European hedgehog, enteritis, chaphamaparvovirus, parvovirus

## Abstract

Starting from June 2022, increased mortality associated with enteric signs was reported in European hedgehogs (*Erinaceus europaeus*) recovered at a regional wildlife rescue center, in Apulia, Italy. Cases of enteric disease were observed until the end of the breeding season, despite increased biosafety measures. A novel parvovirus was identified using metaviromic, and parvovirus-like particles were observed in the stools on electron microscopy observation. The virus was detected in the fecal samples of all the animals tested (*n* = 9) and in the internal organs (liver, spleen, and kidney) of three out of nine animals using a specific quantitative assay. In the full-length genome, the parvovirus was closely related (90.4% nt) to a chaphamaparvovirus identified in an Amur hedgehog (*Erinaceus amurensis*) in Asia and to chaphamaparvoviruses (≤ 70% nt) detected in bats and rodents. Since chaphamaparvoviruses are considered as pathogen in rodents, it will be important to investigate the pathogenic role, if any, of these parvoviruses in hedgehogs.

**IMPORTANCE:**

European hedgehogs (*Erinaceus europaeus*) are common in Europe. This species has been shown to harbor occasionally zoonotic pathogens, including bacteria, fungi, and viruses. Exploring the virome of wildlife animals is important for animal conservation and also to assess zoonotic risks. Our metaviromic investigation identified a novel parvovirus from an outbreak of enteritis in European hedgehogs housed in a wildlife rescue center, extending the spectrum of potential viral pathogens in this species.

## OBSERVATION

European hedgehogs (*Erinaceus europaeus*) are common in Europe (1) and can be found in various habitats. Although possessing European hedgehogs is illegal in most Western countries, including Italy, often juvenile or injured individuals can be retrieved and temporarily or permanently kept in households. This has generated concerns about the potential of zoonotic diseases transmissible from hedgehogs to humans, caused by viral, bacterial, and fungal pathogens (2), including Middle East respiratory syndrome (MERS)-like betacoronaviruses (3). In this work, we describe an enteric disease associated with increased mortality in immature weaned hedgehogs housed in a rescue center in Southern Italy in the 2022 breeding season.

In the period June–July 2022, increased mortality was reportedly observed in orphaned weaned European hedgehogs, aged between 1 and 5 months, rescued at the Regional Wildlife Rescue Centre of Bitetto, prefecture of Bari, Apulia, Italy. Overall, on a year-to-year basis, mortality in this time span in immature hedgehogs increased from 16% (7/44) in 2021 to 53% (19/36) in 2022.

At the beginning of June 2022, three subjects (considered the initial case), hospitalized in the pre-release acclimatization enclosure with seven other hedgehogs, died simultaneously during the night without overt clinical signs. Necropsy was carried out on the carcasses revealing inflammation of the small intestine with thickening of the walls, congestion of the mucosa, and the intestinal loops overextended due to gas accumulation. Congestion of the liver and spleen was also observed. Parasitological examinations carried out by flotation and bacteriological analysis of the intestinal samples were not conclusive.

Pooled stool samples from the initial case were subjected to a sequence-independent amplification protocol (4), and the libraries were sequenced using Minion (Oxford Nanopore Technology, ONT) platform. FASTq data were analyzed using WIMP tool on the cloud-based analysis platform EPI2ME (ONT). About 85% of the sequence reads were of bacterial origin, and 64% of the bacterial reads were mapped to *Bacteroides fragilis*. Less than 3% of the reads were of viral origin, mostly from bacteriophages. Using the metaviromic pipeline of Genome Detective (5), parvovirus-related contigs were also generated, and the genome of strain ITA/2022/265 (GenBank accession nr OQ919797) was reconstructed by combining 5′ RACE (rapid amplification of cDNA ends) protocols (6) and a primer walking strategy with specific primers designed to close the gaps among non-contiguous sequences. The genome was 4.4 kb in length and included three open reading frames: accessory protein p15 and non-structural (NS) and capsid (VP) proteins (Table 1). Genome-wide virus showed ≤70% nucleotide (nt) identity to parvoviruses detected in bats and rodents, including parvoviruses detected in laboratory rodents and associated with chronic tubulointerstitial nephropathy and kidney fibrosis (7) and recently classified within the genus *Chaphamaparvovirus*, Hamaparvovirinae sub-family (8).

**TABLE 1:** Features of the complete genome of hedgehog chaphamaparvovirus sequenced in this study^*a*^

	ITA/2022/hedgehog/265 (OQ919797)						
	Complete genome	p15		NS		VP	
Genomic features	nt	nt	aa	nt	aa	nt	aa
	4,338	411	136	1,992	663	1,515	504
Identity to other parvoviruses (%)							
Hedgehog chapparvovirus 6/CHN/2020/hedgehog/HeN-F2 (OM451162)	90.4	95.6	97.8	90.4	91.9	88.1	97.0
Desmodus rotundus parvovirus/BRA/2010/bat/DRA25 (NC_032097)	67.7	66.4	55.6	69.8	60.8	68.0	62.8
Murine chapparvovirus/USA/2014 /mouse/Q052_1676 (MF175078)	67.1	66.7	58.1	70.1	62.0	67.9	61.1

^*a*^ nt, nucleotides; aa, aminoacids; p15, accessory protein; NS, non-structural protein; VP, capsid protein.

**Fig 1:**
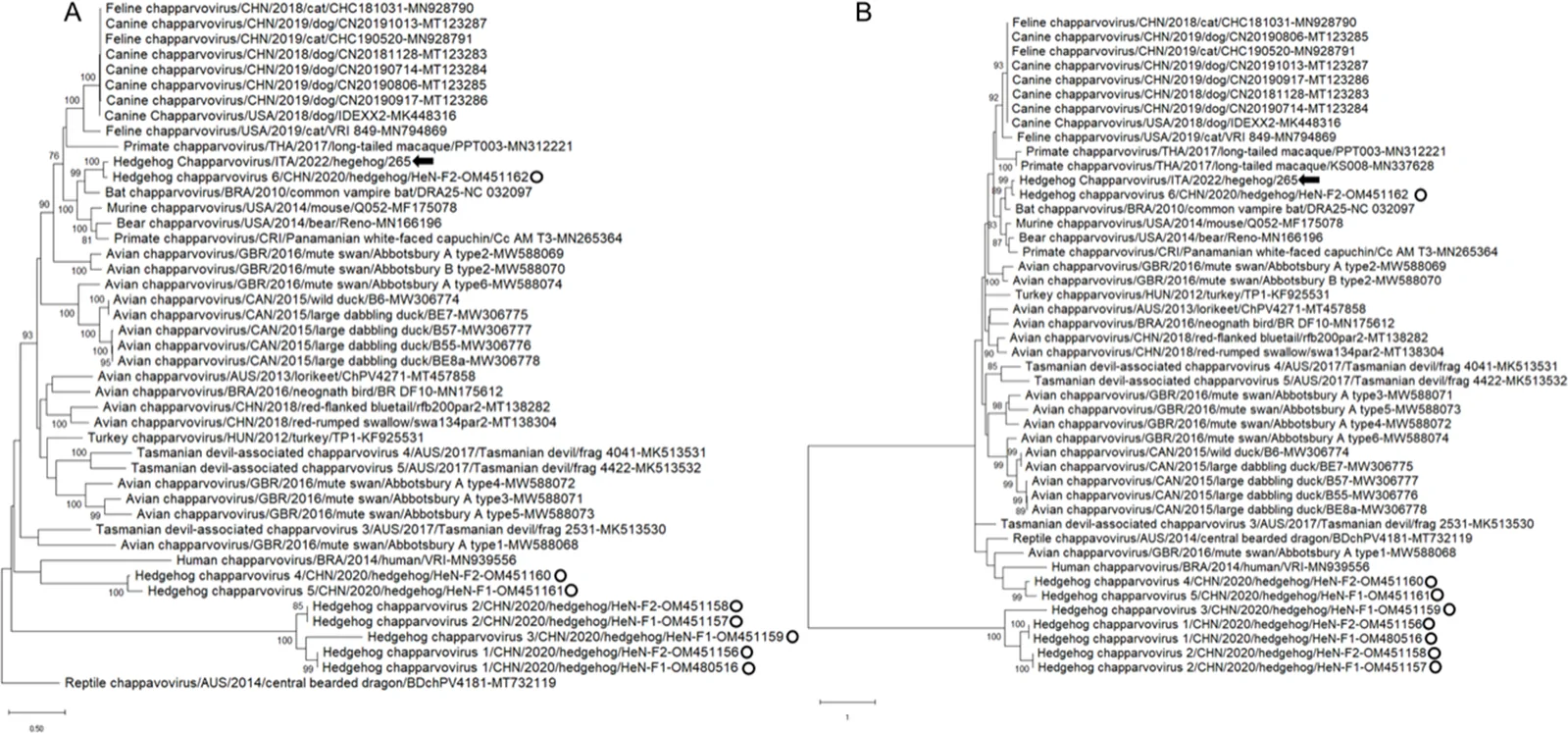
Maximum likelihood phylogenetic trees of chaphamaparvovirus identified in this study and reference strains recovered in the GenBank database. Partial NS (1,531 nt) (**A**) and VP (379 nt) (**B**) sequence-based phylogenetic trees were reconstructed using Tamura-Nei model (four parameters) with a gamma distribution. A total of 1,000 bootstrap replicates were used to estimate the robustness of the individual nodes on the phylogenetic tree. Bootstrap values greater than 75% were indicated. Black arrows indicate the European hedgehog (*Erinaceus europaeus*) strain detected in this study. White circles with black border indicate chaphamaparvoviruses previously identified in amur hedgehogs (*Erinaceus amurensis*). The numbers of nucleotide substitutions are indicated by the scale bar.

However, when re-analyzing the data during the writing of this report, the closest match (90.4% nt at the genome level) was found to a parvovirus identified in Amur hedgehog (*Erinaceus amurensis*) in a large metaviromic investigation in game animals from China (9) (Table 1). NS and VP nucleotide sequences-based phylogenetic analyses confirmed the close relatedness of strain ITA/2022/hedgehog/265 to Amur Hedgehog and to chaphamaparvoviruses detected from bats and rodents (Fig. 1).

**Fig 2:**
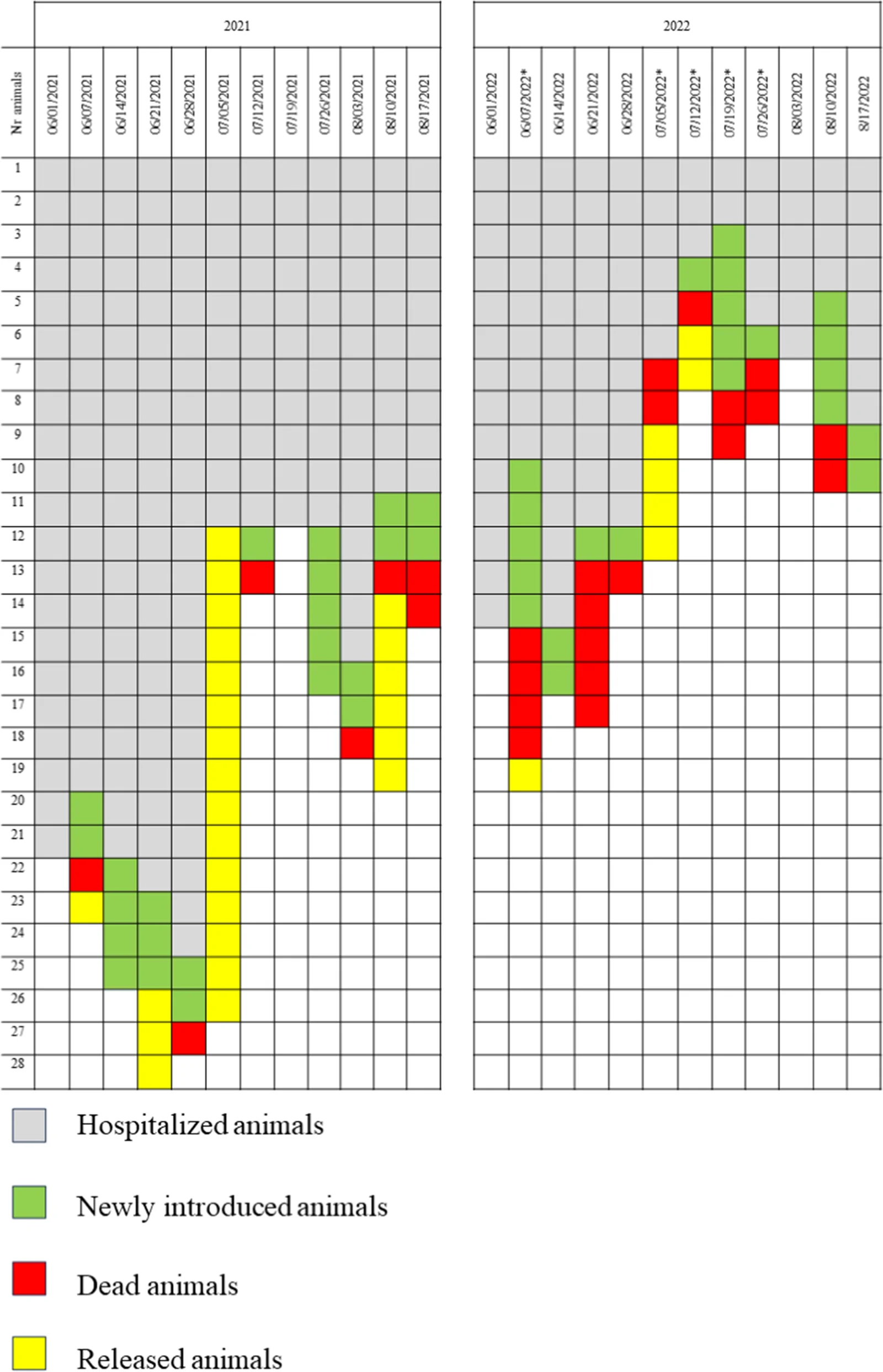
Dynamics (introduced, released, and dead animals) of hedgehog population in the period June to mid-August of 2021 and 2022 at the Regional Wildlife Rescue Centre, Bitetto, Apulia, Italy. Asterisks indicated the collection of samples.

Three other animals of the initial case group died at different time points (Fig. 2). Also, an additional 14 animals died until the end of July. Apparently, death occurred with a similar cohort of clinical signs in most animals. The subjects were all hospitalized for at least 7 days before the onset of clinical signs and initially presented a slight decrease in appetite, evolving into complete anorexia within a few days. At the same time, gastrointestinal signs appeared with the production of semi-solid, dark red, fetid feces. Some animals experienced respiratory signs with sneezing and mild serous nasal discharge. Again, parasitological examinations were negative, while bacteriological (cultural) examinations were inconclusive. Broad-spectrum antibiotic therapy with injectable enrofloxacin (10 mg/kg bid) was immediately administered at the onset of clinical signs. Forced feeding was applied in anorexic animals. Death occurred within 4–6 days of the onset of clinical signs in all the animals with a case fatality rate of 100%.

After the initial case of enteric disease, all the new hedgehogs arriving at the center were housed individually in rabbit cages under quarantine, and the biosafety measures were increased. Deep cleaning and disinfection of the enclosures with sodium hypochlorite were carried out routinely. The outbreak died out with the end of hedgehogs breeding season. After mid-August, the number of new immature subjects admitted to the rescue center drastically decreased.

In total, samples from nine necropsied hedgehogs were frozen and available for analysis. To detect and quantify parvovirus load in samples from the nine animals, we used a quantitative PCR (qPCR). Forward (5′-GGCGTTTCTGTACCAAAGAGGAA-3′) and reverse (5′-GCATTTGCAGCGATGTTGACTAG-3′) primers and probe (5′-FAM-TGCATGATACTACCTTTCATTGCAGA-BHQ1-3′) were designed to amplify a 119-nt segment of the NS1 gene, in a 15-µL reaction master mix iTaq Universal Probes Supermix (Bio-Rad Laboratories SRL, Segrate, Italy) containing 0.6 µM of each primer and 0.2 µM of probe. Thermal cycling consisted of activation of iTaq DNA polymerase at 95°C for 3 min and 42 cycles of denaturation at 95°C for 10 s and annealing-extension at 60°C for 30 s. The virus was detected in the stools of all animals and in the internal organs (liver, kidney, and spleen) of 3/9 tested animals in this qPCR. The viral loads ranged from CT = 23.37 to 39.66

**Fig 3:**
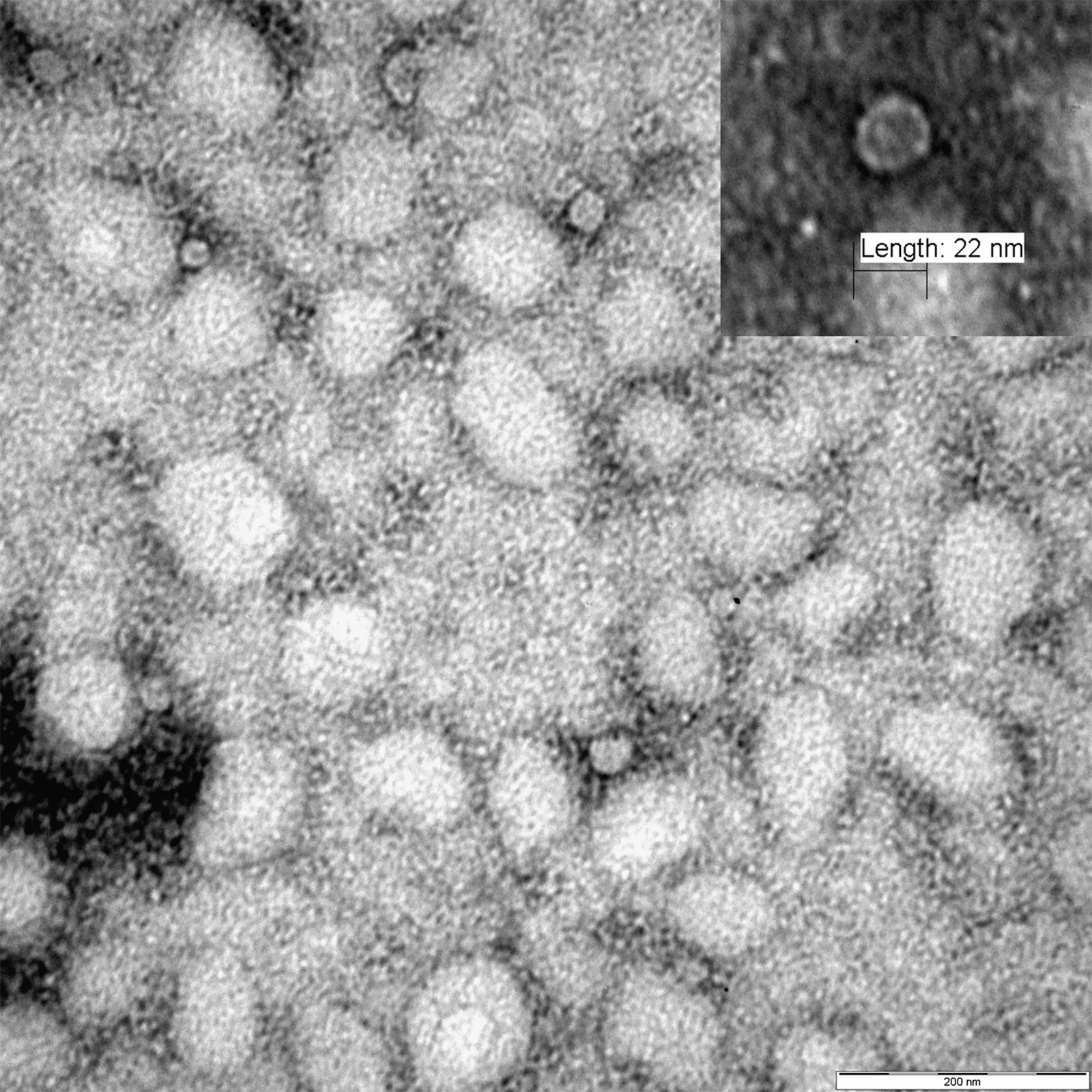
Electron microscopy observation of chaphamaparvovirus-positive stools. Negative staining microphotograph of 23–28 nm rounded particles in the intestinal content. Bar = 22 nm.

Fecal samples with high viral load (CT = 24.89) were processed for EM observation (10). Parvovirus-like particles were observed in the stools (Fig. 3), although the virus was not aggregated with a serum specific for canine parvovirus and with a serum obtained from an adult hedgehog.

Based on deep sequencing analysis of the fecal samples from the initial case, an abundance of *B. fragilis* DNA was observed. Sub-populations of *B. fragilis*, a minor component of human and animal microbiome, may overgrow and produce entero-toxins, causing enteric disease, chiefly in young individuals (11, 12). However, *in silico* analysis did not allow for retrieving sequence reads of the metalloprotease entero-toxin gene in our data set. Also, since information on the bacteriome of hedgehogs is not available, it is not possible to interpret these findings correctly.

In conclusion, we detected a novel parvovirus in European hedgehogs with fatal enteric disease. A limitation of this study was that Kock’s postulates were not fulfilled, and it was not possible to demonstrate a clear association between the increased mortality observed in the rescue center and the hedgehog parvovirus. Chaphamaparvoviruses can induce inclusion body nephropathy and kidney fibrosis in mice (7, 13). Also, chaphamaparvoviruses have been detected at high prevalence in a multi-facility feline shelter during an outbreak of diarrhea and vomiting (14), and in lung, liver, and brain samples collected from bearded dragons showing respiratory or neurological symptoms (15), hinting to a possible pathogenic role for these parvoviruses. Herein, the virus was detected in all nine animals in the gastro-intestinal tract, while it was variously detected in the kidneys, spleen, liver, heart, and lungs, suggesting the possibility of either extra-intestinal target organs or of viremic phases during the course of the infection.

Exploring the virome of wildlife animals is now recognized as a priority in terms of animal conservation and also in the perspective of One Health principles.

## Data Availability

The complete genome sequence of strain ITA/2022/265 was deposited in the GenBank database under accession OQ919797.
